# Triglyceride–Glucose Index May Predict Renal Survival in Patients with IgA Nephropathy

**DOI:** 10.3390/jcm11175176

**Published:** 2022-09-01

**Authors:** Aiya Qin, Jiaxing Tan, Siqing Wang, Lingqiu Dong, Zheng Jiang, Dandan Yang, Huan Zhou, Xiaoyuan Zhou, Yi Tang, Wei Qin

**Affiliations:** 1 West China School of Medicine, Sichuan University, Chengdu 610041, China; 2 Department of Nephrology, West China Hospital of Sichuan University, Chengdu 610041, China; 3 West China School of Public Health, West China Forth Hospital, Sichuan University, Chengdu 610093, China

**Keywords:** triglyceride glucose index, TyG index, IgA nephropathy, renal survival

## Abstract

**Background:** The triglyceride–glucose (TyG) index is a simple, novel and reliable surrogate marker of insulin resistance. However, evidence for the prognostic impact of an elevated TyG index on IgA nephropathy (IgAN) is limited. Therefore, we evaluated the relationship between the TyG index and the risk of renal progression in IgAN. **Method:** This cohort study involved biopsy-proven IgAN between January 2009 and December 2018 in West China Hospital, in which patients were assigned to two groups based on the cut-off value of TyG using receiver operating characteristic (ROC) curves. A 1:1 matched-pair analysis was established to optimize the bias in IgAN by propensity score matching (PSM). The TyG index was calculated as ln [fasting triglyceride (mg/dL) × fasting glucose (mg/dL)/2]. The composite endpoint was defined by eGFR decreased ≥50% of the baseline level, end-stage kidney disease (ESKD), renal transplantation and/or death. Univariable and multivariable Cox proportional hazard models were applied to confirm the predictive value of the optimal marker. **Results:** Before PSM, a total of 1210 participants were ultimately included. During a median follow-up period of 55.8 months (range 37.20–79.09 months), 129 participants progressed to the composite endpoint (10.7%). After PSM, 366 patients were enrolled in the matched cohort, of whom 34 (9.3%) patients reached the endpoints. Based on the cut-off value of the TyG index, patients were divided into the low TyG index group (TyG ≤ 8.72, n = 690) and the high TyG index group (TyG > 8.72, n = 520). Further analysis demonstrated that a higher TyG index was significantly associated with a higher risk of reaching composite endpoints in IgAN patients in both the unmatched and matched cohorts (before PSM: HR 2.509, 95% CI 1.396–4.511, *p* = 0.002; after PSM: HR 2.654, 95% CI 1.299–5.423, *p* = 0.007). **Conclusion:** A high TyG index is associated with a higher risk of renal progression.

## 1. Background

IgA nephropathy (IgAN) is the most common primary glomerulonephritis (GN) worldwide and is characterized by histopathological criteria of mesangial codominant IgA staining on renal biopsy [1]. However, the pathogenesis remains complex and uncertain, with up to 1 in 4 patients experiencing end-stage kidney disease (ESKD) within 20 years of diagnosis [2]. An effective screening strategy is essential for identifying high-risk groups and reducing the incidence rate of ESKD.

Insulin resistance (IR), an early metabolic complication characterized by impaired responses to insulin in organs and tissues, is strongly predictive of CVD (cardiovascular disease) [3]. The “gold standard” for the diagnosis of IR is the hyperinsulinaemic-normoglycaemic clamp test, which is rarely performed in epidemiological investigations of large sample populations because of its expense and complex procedure [4,5]. The triglyceride–glucose (TyG) index, first used in 2008 by Simental-Mendia et al. in an apparently healthy population, has recently been recognized as a reliable surrogate biomarker of IR [6]. It has shown the advantages of being more convenient and easily accessible in clinical practice than plasma insulin in the homeostasis model assessment of IR [7]. Epidemiological and basic science studies have shown that the elevation of the TyG index correlates well with atherosclerosis, cardiovascular risk, progression of chronic kidney disease (CKD), and renal dysfunction [3,8,9,10,11,12], all of which are prevalent all over the world. A previous study showed that glucose metabolism and dyslipidemia were common in IgAN patients, and IR seemed to be associated with the development of kidney dysfunction in IgAN [13,14,15]. However, it is unknown whether the triglyceride–glucose (TyG) index is associated with the risk of renal progression in Chinese IgAN patients. Thus, the present study was conducted to explore the underlying correlation between the TyG index and IgAN.

## 2. Materials and Methods

### 2.1. Study Population

**Figure 1:**
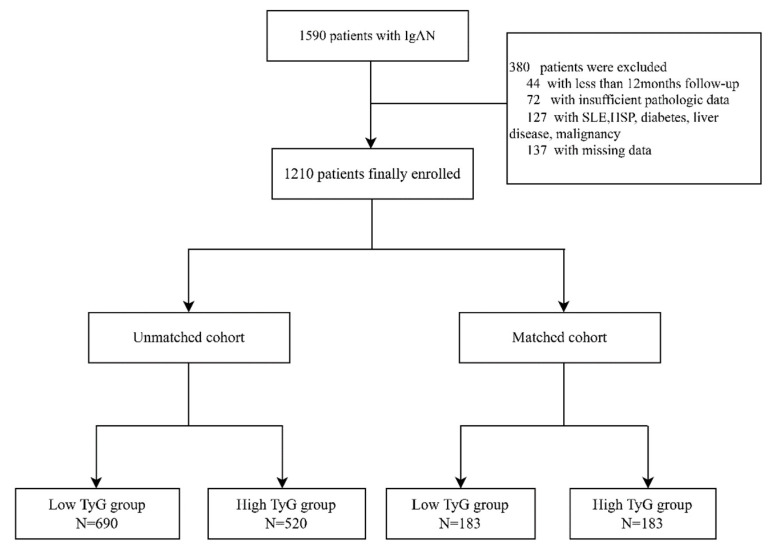
Study profile. Abbreviations: IgAN, IgA nephropathy; SLE, systemic lupus erythematosus; HSP, Henoch–Schönlein purpura.

The study initially included 1590 patients with IgAN diagnosed by renal biopsy at West China Hospital of Sichuan University between January 2009 and December 2018. Among these patients, 72 patients without sufficient pathologic data or renal biopsies containing fewer than eight glomeruli, 137 individuals missing key clinical data during follow-up and 44 subjects with less than 12 months of follow-up before reaching the endpoint were excluded from the study. In addition, we also excluded 127 patients with systemic lupus erythematosus (SLE), Henoch–Schönlein purpura (HSP), diabetes, liver disease, or malignancy. The population before and after adjustment for propensity scores were displayed as the unmatched and matched cohorts, respectively. Ultimately, 1210 patients in the unmatched cohort and 386 patients in the matched cohort were enrolled in our following study (Figure 1). All patients were followed up in the outpatient clinic at least every 1–3 months after the kidney biopsy. This research project follows the Helsinki Declaration and was approved by the ethics committee of West China Hospital, Sichuan University (2019-33).

### 2.2. Clinical and Pathological Data Collection

Baseline clinical data were collected at the time of kidney biopsy as well from the hospital’s electronic medical records system, including age, sex, height, weight, body mass index (BMI), smoking status, medication history, systolic and diastolic blood pressure (SBP, DBP), fasting blood glucose (FPG), serum albumin (ALB), hemoglobin (Hb), serum creatinine (Cr), uric acid (UA), and estimated glomerular filtration rate (eGFR). Renal pathology was reviewed by expert renal pathologists. The histological lesions were classified according to Oxford classification scores (MEST-C, M: mesangial hypercellularity; E: endocapillary hypercellularity; S: segmental glomerulosclerosis; T: tubular atrophy/interstitial fibrosis, and C: crescent) [16].

### 2.3. Treatment and Definitions

The treatment was mainly based on KDIGO guidelines according to the clinical and pathological features of the patients [17]. Patients in the supportive care group (SC) only received an optimal dose of an angiotensin-converting enzyme inhibitor (ACEI) or an angiotensin receptor blocker (ARB). Patients in the corticosteroid group (CS) received optimal ACEIs/ARBs plus corticosteroids (0.5–1 mg/kg prednisone daily and tapering gradually within 6–8 months). Immunosuppressant therapy (IT) included cyclophosphamide (2 mg/kg daily for 3 months), cyclosporine (3–5 mg/kg daily for 6–8 months), mycophenolate mofetil (1–2 g daily for 6–8 months), or tacrolimus (0.03–0.05 mg/kg daily for 6–8 months) [13]. The TyG index was calculated by using the following formula: Ln [TG (mg/dL) × fasting glucose (mg/dL)/2] [18]. The eGFR was calculated using the Chronic Kidney Disease Epidemiology Collaboration (CKD-EPI) equation [19]. Anemia was defined as a hemoglobin concentration lower than 120 g/L in men or lower than 110 g/L in women. Hypoalbuminemia was defined as albumin <30 g/L. Hyperuricaemia was defined by cut-off values of 420 μmol/L (7 mg/dL) and 360 μmol/L (6 mg/dL) for women [20]. There are many group methods of data handling in statistics, such as receiver operating characteristic curves (ROCs) and terstiles [7,21]. In our study, patients were divided into two groups by the optimal cut-off value of the TyG index, which was used by the Youden index calculated by carrying out the ROC curve.

### 2.4. Outcomes

The combined endpoints of renal outcome were eGFR decreased ≥50% of the baseline level, ESKD, renal transplantation and/or death. ESKD was defined as eGFR ≤15 mL/min/1.73 m^2^ or maintenance renal replacement treatment.

### 2.5. Statistical Analysis

Normally distributed continuous variables are expressed as the mean ± standard deviation (SD) and were compared using Student’s *t* test. Non-normally distributed continuous data were expressed as medians and interquartile ranges and compared using the Mann–Whitney U test. Dichotomous data are presented as numbers and percentages and were compared using the χ^2^ test. Spearman’s correlation analysis was used to identify factors potentially correlated with the TyG index. Spearman’s correlation analysis was used to analyze the correlations between the TyG index and serum biochemical parameters. Binary logistic regression analyses were employed to assess whether a high TyG index was a risk factor for more severe clinicopathological manifestations. Univariate and multivariate Cox proportional hazard models were used to evaluate the effect of risk factors on kidney endpoints. Kaplan–Meier survival curves were constructed to assess the predictive value for renal survival. A 1:1 propensity score match (PSM) was then carried out to eliminate significant differences at baseline. Analyses of (ROCs) were undertaken to determine the sensitivity and specificity of the TyG index for predicting renal progression. The analysis was performed using SPSS software (version 26.0; IBM Corp., Armonk, NY, USA), and a two-tailed *p* < 0.05 was considered statistically significant.

## 3. Results

### 3.1. Characteristics of Study Participants

**Figure 2:**
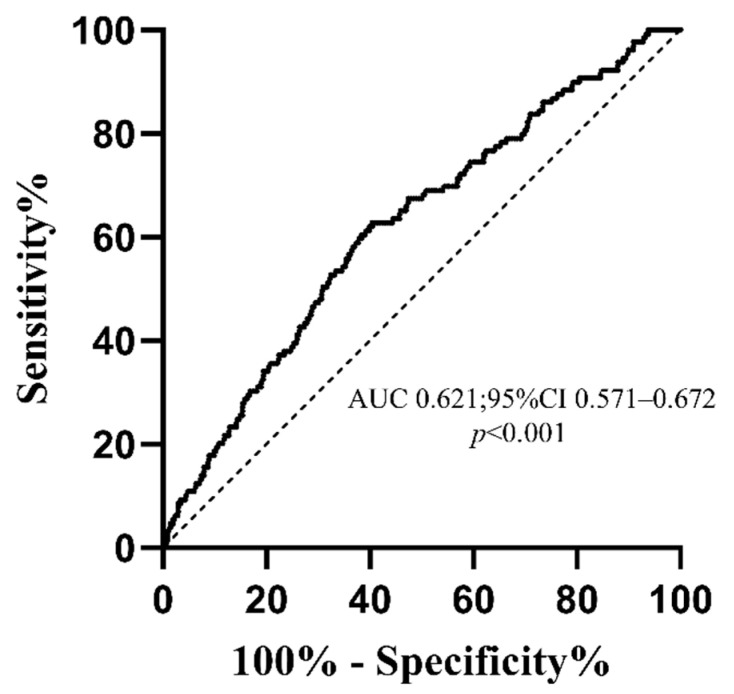
The ROC curve of the TyG index for the prediction of composite endpoints in IgAN patients. Abbreviations: AUC, area under the ROC curve.

In the unmatched cohort, a total of 1210 participants with IgAN were finally included in this retrospective analysis, of whom 129 (10.7%) patients reached the endpoints. The median age of the patients was 32 (25–41) years, and 537 (44.4%) patients were men. The median baseline eGFR was 94.2 (67.2–117.7) mL/min/1.73 m^2^, and the median 24-h proteinuria was 1.35 (0.72–2.85) g/d. The relative ability of TyG indices to identify patient progression to composite endpoints in IgAN patients is illustrated in Figure 2. The AUC of the TyG index was 0.621 (95% confidence interval [CI] 0.571–0.672), and the cut-off value based on the Youden index for the prediction of the composite endpoint was 8.72. According to the optimal cut-off values of the TyG index, patients were divided into the low TyG group (TyG ≤ 8.72, n = 690) and high TyG group (TyG > 8.72, n = 520). Compared with the low TyG group, patients in the high TyG group were significantly more likely to be smokers and had a higher proportion of males (49.6% vs. 40.4%, *p* = 0.002), hypertension (34.6% vs. 21.0%, *p* < 0.001), anemia (16.3% vs. 12.0%, *p* < 0.001), and hyperuricemia (47.1% vs. 30.6%, *p* < 0.001). Additionally, the high TyG group people had higher SBP (128 (119–140) vs. 123 (113–135) mmHg, *p* < 0.001), DBP (84.5 (77.0–93.0) vs. 80 (73–89) mmHg, *p* < 0.001), and higher Cr (91.4 (71.0–121.0) vs. 76.3 (61.6–98.9) umol/L, *p* < 0.001) and higher triglyceride (2.19 (1.80–3.00) vs. 1.08 (0.84–1.30) mmol/L, *p* < 0.001), FPG (5.1 (4.7–5.6) vs. 4.7 (4.4–5.1) mmol/L, *p* < 0.001), and proteinuria (2.00 (1.00–3.36) vs. 1.01 (0.57–2.04) g/d, *p* < 0.001). In terms of the pathological lesions, the low TyG group showed a lower incidence of S (64.4% vs. 57.7%, *p* = 0.018), T (25.2% vs. 15.4%, *p* < 0.001), and C (25.6% vs. 21.2%, *p* = 0.073). With respect to treatment, a large proportion of immunosuppression and/or steroids and less supportive treatment only (*p* < 0.001) therapy were shown in the high TyG index group.

To eliminate the difference between the two groups, patients (183 in each group) in the matched cohort were enrolled after PSM at a 1:1 ratio, in which 34 (9.3%) patients reached the composite endpoints. There were no differences in clinicopathological manifestations and treatments except serum conjugated and unconjugated TyG levels. The clinical characteristics of participants grouped by the cut-off value of the TyG index before and after PSM are shown in Table 1.

### 3.2. Correlation of the TyG Index with IgAN

Pearson’s or Spearman’s rank correlation analysis was performed to explore the relationship between the TyG index and clinical baseline data. As presented in Table 2, the TyG index was positively correlated with BMI (r = 0.350, *p* < 0.001) as well as proteinuria (r = 0.331, *p* < 0.001) and UA (r = 0.244, *p* < 0.001) levels and negatively correlated with eGFR (r = −0.262, *p* < 0.001) and Alb (r = −0.095, *p* < 0.001). Histopathologically, logistic regression analysis (Table 3) revealed that there was statistical significance in S_1_ and T_1-2_ lesions, with odds ratios (ORs) of 1.329 (1.051–1.680, *p* = 0.018) and 1.855 (1.393–2.471, *p* < 0.001) and slight statistical significance of C1-2/C0 (1.281 (0.979–1.675, *p* = 0.071), respectively. Notably, the high TyG index group patients tended to have hypertension (OR 1.990, 95% CI 1.538–2.574, *p* < 0.001), be smokers (OR 1.990, 95% CI 1.538–2.574, *p* < 0.001), and have worse renal function (eGFR < 60 mL/min.1.73m^2^: OR 2.120, 95% CI 1.594–2.819, *p* < 0.001).

### 3.3. The TyG Index for Predicting Renal Survival

**Figure 3:**
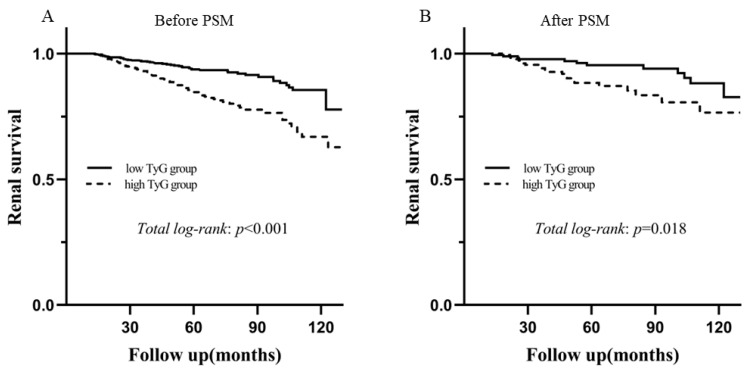
Kaplan‒Meier analysis for renal survival between the high TyG group and the low TyG group in the unmatched and matched cohorts. (**A**) The unmatched cohort and (**B**) the matched cohort. Abbreviation: PSM, propensity score match.

During the median follow-up of 55.8 months (range 37.20–79.09 months), 129 patients progressed to the composite endpoint before PSM, accounting for 10.7% of the total number. As shown by Kaplan–Meier analysis, there was a significant difference in long-term outcomes between the two groups regardless of the unmatched cohort (*p* < 0.001) and matched populations (*p* = 0.018) according to the TyG level, as shown in Figure 3. The univariate analysis showed that a TyG index >8.72 was significantly associated with a higher risk of the incidence of the composite endpoint [before PSM: hazard ratio (HR) 2.483, 95% CI 1.736–3.551, *p* < 0.001; after PSM: HR 2.295, 95% CI 1.131–4.657, *p* = 0.021]. As shown in Table 4, three models were used for the multivariate Cox proportional hazards regression analysis. After adjusting for age, sex, Oxford classification of IgAN (MEST-C scores), BMI, SBP, DBP smoking, eGFR <60 mL/min.1.73m^2^, proteinuria, URBC, anemia, hypoalbuminemia, hyperuricemia, and treatments (Model 3), a high TyG index was an independent risk factor for the progression of IgAN to the composite endpoint (before PSM: HR 2.509, 95% CI 1.396–4.511, *p* = 0.002; after PSM: HR 2.654, 95% CI 1.299–5.423, *p* = 0.007).

## 4. Discussion

IgAN is the most common primary GN worldwide, but the critical mechanism is not yet clear [2]. For decades, increasing evidence has shown that IR, a well-established hallmark of metabolic disorders and systemic inflammation, may play an important role in the progression of renal impairment [22,23], whereas the relationship between TyG and IgAN progression remains unknown.

The findings of this study showed that the TyG index has a strongly positive relationship with IgAN progression. Correlation analysis showed that the TyG index was negatively correlated with eGFR and positively correlated with proteinuria. Histopathologically, the high TyG group displayed more severe pathological lesions (endocapillary proliferation and crescents). To simulate randomization and improve balance on baseline variables, all patients were assigned a 1:1 PSM identifying age, gender and treatment modality with the occurrence of TyG as the dependent variable to adjust for the observed characteristics of nonrandomly assigned patients. Overall, only TG and FPG showed significant differences between the two groups. Interestingly, the result strengthened the association between the high TyG index (TyG > 8.72) and the renal outcome of IgAN. Consistent with these findings, previous epidemiologic studies have identified that TyG is positively connected with deterioration of renal function in diabetic nephropathy [24] and contrast-induced nephropathy [11], but no relevant literature confirms and compares a significant correlation between TyG indices and IgAN thus far.

The molecular mechanism through which the TyG index aggravates glomerular sclerosis in IgAN has not been identified. The correlation between the TyG index and renal progression of IgAN can be attributed to the pathogenetic association between IR and IgAN. Thus, the potential mechanisms underlying IR and IgAN progression may be described as follows. IR can induce glucose metabolism imbalance, causing excessive glycosylation, which can promote smooth muscle cell proliferation, collagen crosslinking, and collagen deposition, as well as triggering inflammation and oxidative stress [25]. In addition to its role in hyperglycaemia, IR plays an important role in hyperlipidaemia, which induces oxidative stress and apoptosis, causing the initiation of atherosclerosis and podocyte injury via fibrosis, inflammation and apoptosis [26,27]. Moreover, mounting evidence has revealed that IR can induce an increased production of glycosylated products and free radicals, leading to nitric oxide (NO) inactivation. The abnormal secretion of NO could change renal hemodynamics, which results in damage to the vascular endothelium and causes endothelium-dependent vasodilation [28].

The hyperinsulinaemic-euglycaemic clamp technique is the gold standard to assess IR, which is costly and time-consuming and is likely to cause significant bias owing to insulin measurements [4,5]. In this regard, the TyG index, as a reliable marker of IR and metabolic syndrome, can be easily calculated because required values can be obtained from routine laboratory tests in a minimally invasive, inexpensive, and convenient way. Moreover, it may provide a potential target for intervention in the prevention of renal progression. As such, we recommend the application of TyG in risk assessments for IR in clinical practice and future epidemiologic studies.

In our study, patients were grouped by the optima cut-off value of 8.72 for the TyG index with an AUROC of 0.621, similar to previous studies in which the TyG had an AUROC of 0.644 and 0.607 for the prediction value, respectively [29,30]. There were also studies using the tertiles. Qin et al. revealed that patients with a TyG index >8.99 tended to have higher rates of nephrolithiasis recurrence [7]. Our value was close to those of previous studies. Even so, with an AUROC of 0.621, the predictive performance needs to be improved. This might be explained by the fact that mild renal disease progresses slowly and the short follow-up period.

Nevertheless, based on current studies, there are several limitations in this study that need to be addressed. First, this was a single-center study with a relatively short follow-up period. Second, the change in the TyG index in the follow-up period was missed, which may have an impact on the prognosis. Moreover, due to the retrospective nature, some potential confounding factors, such as data on lipid-lowering agents, were lacking. Last, therefore, high-quality prospective studies with large samples and longer follow-up periods are needed.

## 5. Conclusions

The TyG index, a surrogate marker of insulin intolerance and metabolic syndrome, was associated with severe clinicopathological manifestations and could be used as an independent risk factor for IgAN progression in a minimally invasive and inexpensive manner.

## Figures and Tables

**Table 1:** Baseline characteristics of IgAN patients.

Variable	Unmatched Cohort			Matched Cohort		
	Low TyG	High TyG	*p* Value	Low TyG	High TyG	*p* Value
Numbers (%)	690 (57.0)	520 (43.0)		183	183	
Age (year)	30 (24–39)	35 (27–43)		34.0 (27.0–42.0)	34.0 (26.0–42.0)	0.836
Gender (male, %)	279 (40.4)	258 (49.6)	0.002	95 (51.9)	90 (49.2)	0.676
Hypertension (%)	145 (21.0)	180 (34.6)	<0.001	52 (28.4)	45 (24.6)	0.477
SBP (mmHg)	123 (113–135)	128 (119–140)	<0.001	125.0 (116.0–138.0)	124.0 (117.0–136.0)	0.953
DBP (mmHg)	80 (73–89)	84.5 (77.0–93.0)	<0.001	81.0 (75.0–92.0)	82.0 (75.0–89.0)	0.915
BMI (kg/m^2^)	21.5 (19.6–24.0)	23.7 (21.1–26.6)	<0.001	22.2 (20.3–25.0)	20.5 (23.1–25.7)	0.249
Smoking (%)	97 (14.1)	108 (20.8)	0.002	35 (19.1)	33 (18.0)	0.893
**CKD stages (%)**			<0.001			0.613
Stage 1	437 (63.3)	223 (42.9)		86 (47.0)	96 (52.5)	
Stage 2	150 (21.7)	156 (30.0)		58 (31.7)	50 (27.3)	
Stage 3	92 (13.3)	116 (22.3)		35 (19.1)	35 (19.1)	
Stage 4	11 (1.6)	25 (4.8)		4 (2.2)	2 (1.1)	
**Pathologic**						
M1 (%)	522 (75.7)	401 (76.3)	0.585	142 (77.6)	136 (74.3)	0.541
E1 (%)	27 (3.9)	30 (5.8)	0.134	7 (3.8)	4 (2.2)	0.543
S1 (%)	398 (57.7)	335 (64.4)	0.018	109 (59.6)	113 (61.7)	0.748
T1-2/T0 (%)	106 (15.4)	131 (25.2)	<0.001	33 (18.0)	31 (16.9)	0.891
C1-2/C0 (%)	146 (21.2)	133 (25.6)	0.073	48 (26.2)	40 (21.9)	0.392
**Clinical**						
Cr (umol/L)	76.3 (61.6–98.9)	91.4 (71.0–121.0)	<0.001	87.0 (70.0–110.0)	84.4 (68.0–109.0)	0.441
eGFR (mL/min/1.73 m^2^)	103.1 (75.9–121.1)	82.4 (57.9–107.6)	<0.001	86.5 (66.3–112.4)	92.7 (66.3–114.4)	0.484
ALB (g/L)	40.4 (36.8–43.6)	39.9 (35.7–43.3)	0.071	40.0 (35.3–44.0)	41.0 (37.1–44.0)	0.318
TG (mmol/L)	1.08 (0.84–1.30)	2.19 (1.80–3.00)	<0.001	1.1 (0.9–1.3)	2.0 (1.7–2.9)	<0.001
FPG (mmol/L)	4.7 (4.4–5.1)	5.1 (4.7–5.6)	<0.001	4.7 (4.4–5.0)	5.1 (4.7–5.6)	<0.001
Proteinuria (g/d)	1.01 (0.57–2.04)	2.00 (1.00–3.36)	<0.001	1.4 (0.7–2.8)	1.4 (0.9–2.9)	0.544
URBC (/HP)	22.0 (8.0–76.3)	15.0 (5.0–54.0)	0.002	18.0 (6.0–61.0)	15.0 (5.0–54.0)	0.436
Anemia (%)	83 (12.0)	85 (16.3)	0.036	26 (14.2)	25 (13.7)	1.000
Hyperuricemia (%)	211 (30.6)	245 (47.1)	<0.001	70 (38.3)	74 (40.4)	0.748
**Treatment**			<0.001			0.376
supportive care	316 (45.8)	182 (35.0)		64 (35.0)	75 (41.0)	
steroids only	230 (33.3)	192 (36.9)		73 (39.9)	61 (33.3)	
Immunosuppression and/or steroids	144 (20.9)	146 (28.1)		46 (55.4)	47 (25.7)	

Abbreviations: SBP, systolic blood pressure; DBP, diastolic blood pressure; BMI, body mass index; M, mesangial proliferation; E, endocapillary proliferation; S, segmental glomerulosclerosis; T, tubular atrophy or interstitial fibrosis; C, crescents; Cr, serum creatinine; eGFR, estimated glomerular filtration rate; ALB, albumin; TG, triglyceride; FPG, fasting plasma glucose; URBC, urinary red blood cell counts.

**Table 2:** Correlation between TyG index and potential risk factors in the matched cohort.

Variables	Correlation Coefficient (r)	*p* Value
Proteinuria	0.331	<0.001
Hb	0.033	0.253
Alb	−0.095	0.001
BMI	0.350	<0.001
UA	0.244	<0.001
eGFR	−0.262	<0.001

Abbreviations: Hb, hemoglobin; ALB, serum albumin; BMI, body mass index; UA, uric acid; eGFR, estimated glomerular filtration rate.

**Table 3:** Logistics Regression Models for the relationship between TyG index and kidney pathologic lesion and clinical manifestation.

Variables	OR	95%CI	*p* Value
M	1.085	0.829–1.419	0.554
E	1.503	0.882–2.562	0.134
S	1.329	1.051–1.680	0.018
T1-2/T0	1.855	1.393–2.471	<0.001
C1-2/T0	1.281	0.979–1.675	0.071
Hypertension	1.990	1.538–2.574	<0.001
Smoking	1.603	1.185–2.167	<0.002
eGFR < 60 mL/min.1.73 m^2^	2.120	1.594–2.819	<0.001

Abbreviations: M, mesangial proliferation; E, endocapillary proliferation; S, segmental glomerulosclerosis; T, tubular atrophy or interstitial fibrosis; C, crescents.

**Table 4:** The Univariate and multivariate Cox proportional hazards regression models for composite endpoint in patients with IgAN in the unmatched and matched cohort.

TyG Index	Univariant		Model 1		Model 2		Model 3	
	HR (95% CI)	*p*	HR (95%CI)	*p*	HR (95%CI)	*p*	HR (95%CI)	*p*
Before PSM	2.483 (1.736–3.551)	<0.001	1.882 (1.312–2.700)	0.001	2.510 (1.398–4.508)	0.002	2.509 (1.396–4.511)	0.002
After PSM	2.295 (1.131–4.657	0.021	2.391 (1.175–4.864	0.016	2.545 (1.250–5.182)	0.010	2.654 (1.299–5.423)	0.007

Model 1 adjusted age, gender, Oxford classification of IgA (MEST-C scores). Model 2 adjusted age, gender, BMI, SBP, DBP smoking, eGFR < 60 mL/min.1.73 m^2^, proteinuria, URBC, anemia, hypoalbuminemia, hyperuricemia, treatments (SC, CS, IT). Model 3 adjusted covariates in model 1 and model 2.

## Data Availability

Data regarding the study are available upon request to the corresponding author.
